# Salivary Cortisol as a Biomarker for Assessing Fear and Anxiety in Patients with Molar–Incisor Hypomineralization

**DOI:** 10.3390/diagnostics15040489

**Published:** 2025-02-17

**Authors:** Laura-Roxana Contac, Silvia Izabella Pop, Minodora Dobreanu, Madalina Oprica, Septimiu Voidazan, Cristina Ioana Bica

**Affiliations:** 1 Pedodontics Departament, Faculty of Dental Medicine, George Emil Palade University of Medicine, Pharmacy, Science and Technology of Targu Mureş, 540142 Targu Mures, Romania; lauracontac92@gmail.com (L.-R.C.); cristina.bica@umfst.ro (C.I.B.); 2 Ortodontics Departament, Faculty of Dental Medicine, George Emil Palade University of Medicine, Pharmacy, Science and Technology of Targu Mureş, 540142 Targu Mures, Romania; 3 Immunology Laboratory, Center for Advanced Medical and Pharmaceutical Research, George Emil Palade University of Medicine, Pharmacy, Science and Technology of Targu Mureş, 540142 Targu Mures, Romania; minodora.dobreanu@umfst.ro (M.D.); madalina.nedelcu@umfst.ro (M.O.); 4 Epidemiology Department, George Emil Palade University of Medicine, Pharmacy, Science and Technology of Targu Mureş, 540142 Targu Mures, Romania; septimiu.voidazan@umfst.ro

**Keywords:** molar hypomineralization, cortisol, saliva, stress, psychological, dental anxiety

## Abstract

**Background/Objectives**: Molar–incisor hypomineralization (MIH) is a prevalent dental condition characterized by hypomineralized enamel affecting the first permanent molars and incisors. It leads to visible enamel opacities, with varying severity. Children with MIH often experience dental hypersensitivity, which can result in increased dental fear and anxiety, complicating dental treatment. Salivary cortisol, a well-established biomarker of stress, has been used to assess stress levels in various pediatric conditions but has not been extensively studied in MIH. This study aimed to assess salivary cortisol levels as a stress biomarker in children with MIH and compare them to those in children without MIH. **Methods**: Sixty children aged 5–9 years were divided into two groups: 31 with MIH and 29 healthy controls. Salivary cortisol levels were measured using ELISA, and statistical analysis was performed using IBM SPSS software, version 23 The Mann–Whitney test was used for group comparison, and the Kruskal–Wallis test evaluated the correlation between MIH severity and cortisol levels. **Results**: Children with MIH showed significantly higher mean cortisol levels (2.63 ng/mL) compared to controls (0.96 ng/mL), with a *p*-value of 0.0001. A progressive increase in cortisol levels was observed with the severity of MIH, with the highest levels recorded in grade 3 (4.38 ng/mL), in contrast to grade 0 (0.95 ng/mL), with a *p*-value of 0.001. **Conclusions**: Salivary cortisol levels are significantly higher in children with MIH, suggesting that MIH-related stress may contribute to dental anxiety and hypersensitivity. These findings highlight the importance of stress management in pediatric dental care.

## 1. Introduction

Molar–incisor hypomineralization (MIH) is a prevalent dental condition in children, primarily affecting the first permanent molars and incisors with hypomineralized enamel. This results in visible opacities on the enamel that vary in color from white to yellow brown. The global prevalence of MIH varies widely, with reports of the lowest prevalence in China in 2008 (2.8%), a mean prevalence of 9.4% in Europe, and of 14.3% in Romania [1,2].

Despite its recognition as a significant global public health issue, the exact cause of MIH remains unclear. The condition is categorized into four severity grades that help guide treatment decisions: mild, moderate, severe, and atypical restoration. Grade 3, or PEB (post-eruptive enamel breakdown), refers to severe cases characterized by demarcated opacities and post-eruptive loss of dental hard tissue [3,4]. Beyond the cosmetic effects, MIH is often linked to dental hypersensitivity and a heightened susceptibility to caries [5].

In 2022, the European Academy for Pediatric Dentistry (EAPD) proposed an updated guide for practitioners that serves as a valuable tool for evaluating, documenting, and communicating the severity of MIH. This guide aids in treatment planning, monitoring progress, and supporting research. The EAPD guide emphasizes that affected teeth often display sensitivity, varying from a mild reaction to external stimuli to spontaneous hypersensitivity, and MIH-affected molars can be challenging to anesthetize, complicating dental treatment [6]. However, a more commonly used system classifies MIH-related hypersensitivity as follows:

- Grade 0: Absence of hypersensitivity—The tooth shows no sensitivity to stimuli.
- Grade 1: Mild hypersensitivity—The tooth shows reduced sensitivity without significantly affecting daily activities.
- Grade 2: Moderate hypersensitivity—Tooth sensitivity is moderate, causing occasional discomfort during certain activities.
- Grade 3: Severe hypersensitivity—Sensitivity is intense, causing frequent or continuous pain that significantly affects the patient’s daily activities and quality of life [7].

It is well known that children diagnosed with severe MIH exhibit significantly higher levels of dental fear and anxiety compared to children who do not have this enamel defect [8]. The fear is mainly related to the hypersensitivity and pain associated with the affected teeth, which are aggravated during dental procedures [9].

Due to increased fear and anxiety, children with MIH and active caries grafted on enamel defects have avoidant behaviors and reduced cooperation during dental treatments. This presents a challenge for dental practitioners, who may need to apply advanced behavior management techniques.

Possible causes of anxiety may be pain experienced during daily activities such as eating or brushing, previous traumatic experiences during dental procedures caused by MIH-specific hypersensitivity, or anticipation of pain during treatments involving the affected teeth [10].

Studies regarding dental hypersensitivity and behavioral management difficulties in children with MIH are conflicting, with some research indicating that these children exhibit behavioral problems associated with dental anxiety and hypersensitivity, while other studies claim that there are insufficient objective tools to demonstrate that patients with MIH are more difficult to manage in the dental office compared to those without MIH [11,12].

Dental fear and anxiety (DFA) represent intense emotional responses related to dental treatment, often influenced by childhood experiences. Dental fear occurs as a reaction to specific stimuli in the dental office, whereas dental anxiety involves a generalized state of anxiety about possible discomfort. Distinguishing between the two can be difficult, but both can affect dental treatments [13].

Acute stress is defined as the body’s immediate and short-lived physiological response to a situation perceived as threatening or demanding. This type of stress also occurs in dental office anxiety as an emotional and behavioral reaction immediately following the action of a specific, unexpected, or high-intensity factor, such as danger, conflict, or unusual challenge [14]. DFA in response to an acute stressor is characterized by the activation of the sympathetic nervous system and the release of stress hormones, such as adrenaline and cortisol, which prepare the body for the fight-or-flight response [13].

Regarding salivary cortisol as a biomarker, it can be used to objectively assess children’s stress levels as a marker of anxiety and can be a valuable diagnostic tool for oral health. It can serve in the identification, prevention, and optimal management of dental anxiety, involving behavioral management techniques, as well as creating a friendly environment for children and giving them the necessary emotional support [15].

Research on salivary cortisol levels in children during dental treatments has provided important insights into how these procedures impact physiological stress responses. Salivary cortisol is a noninvasive biomarker widely used to assess stress, and its levels can reflect the anxiety experienced by children in dental settings. In 1998, Klingberg et al. found that cortisol levels were higher in children who reported greater dental fear, suggesting a close relationship between subjective anxiety and physiological stress markers [16].

A 2013 study demonstrated that children undergoing dental extractions exhibited significant increases in salivary cortisol levels, indicating elevated stress. Moreover, interventions aimed at reducing dental anxiety, such as cognitive–behavioral techniques or the use of audiovisual distractions, have been shown to effectively lower cortisol levels, thus improving the dental experience for young patients. These findings underscore the importance of managing stress in pediatric dental care to promote better health outcomes and patient cooperation [17,18].

The aim of this study was to determine salivary cortisol levels in children with MIH and to compare these levels with those in children without this condition, based on the hypothesis that salivary cortisol levels, as a biomarker of stress, are significantly higher in children with MIH compared to children without this condition.

## 2. Materials and Methods

This case-control study was carried out between July and September 2024, with the approval of the Ethics Commission (Decision of the Scientific Research Ethics Commission No. 3307 of 12 July 2024), assessing salivary cortisol levels in children with mixed dentition who were patients in the Natural Smile by Dr Pop Dental Clinic in the Transylvania region. The study included children, regular patients of the clinic, aged between 5 and 9 years. The initial group consisted of 70 participants, of which 10 were excluded based on the exclusion criteria outlined below. A total of 60 participants remained in the study, divided into two groups: the study group, which included 32 children diagnosed with MIH of varying degrees of severity, and the control group, which included 28 children without MIH. The protocol for determining the varying degrees of severity of MIH was developed based on the classification of K. Mathu-Muju and J.T. Wright. Mild (Grade 1): Limited demarcated opacities located in non-stress-bearing areas, with mild involvement of the incisors, but no hypersensitivity. Moderate (Grade 2): Enamel defects limited to one or two surfaces without cuspal involvement, and the presence of hypersensitivity. Severe (Grade 3): Post-eruptive enamel breakdown, crown destruction, caries associated with affected enamel, and a history of dental hypersensitivity [19].

The evaluation of salivary cortisol levels was performed using the quantitative competitive ELISA (enzyme-linked immunosorbent assay) method using salivary cortisol ELISA tests by DRG International, Inc., Springfield, NJ, USA. Kit sensitivity: the detection limit for cortisol is 0.082 ng/mL. Kit precision: the intra-assay precision has a coefficient of variation below 8.3%, and the inter-assay precision has a coefficient of variation below 13.6%.

Patient inclusion criteria specified children with mixed dentition, patients with regular dental visits, with known medical history, absence of general chronic or acute pathologies, absence of any medication or dietary supplements in the last month, absence of untreated carious lesions, and with permanent incisors and molars present on the arch.

Exclusion criteria ruled out patients with recent acute illnesses (infections, viruses, trauma), patients with any type of recent medication (including anti-inflammatory or anesthetic), and those with inflammation or any type of intra-oral lesion.

After informing parents and children about the conduct of the study and obtaining consent for the voluntary participation of patients, verbal and written informed consent was obtained from participants and their legal guardians, who were given a detailed explanation of the procedure and its purpose.

Patient preparation:

1. Collection of unstimulated saliva was carried out in the first part of the day, between 9 and 11 a.m., in 1.5 mL Eppendorf containers
2. The patients were scheduled at least 60 min after waking up, after consuming any type of food or drink.
3. Teeth brushing or mouthwash use was avoided for at least 1 h before.
4. A state of physical and emotional rest before harvesting was ensured.
5. The 1.5 mL samples were stored at −80 degrees Celsius in hermetically sealed Eppendorf containers, then slowly thawed to prevent biomarker degradation.

**Figure 1:**
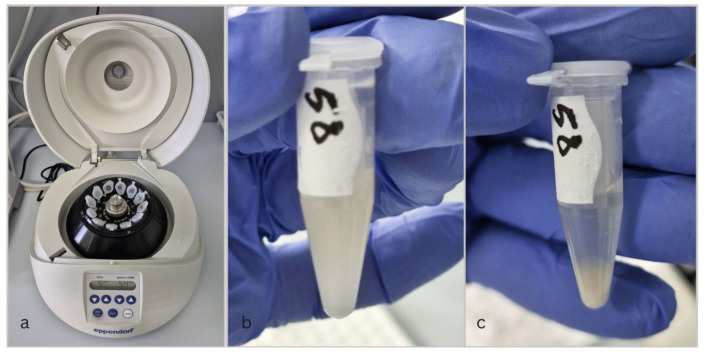
(**a**) MiniSpin Eppendorf centrifuge; (**b**) saliva sample before centrifugation; (**c**) saliva sample after centrifugation.

**Figure 2:**
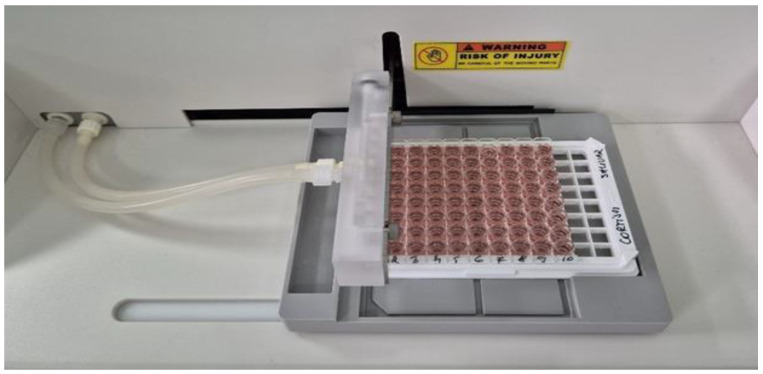
The washing process.

**Figure 3:**
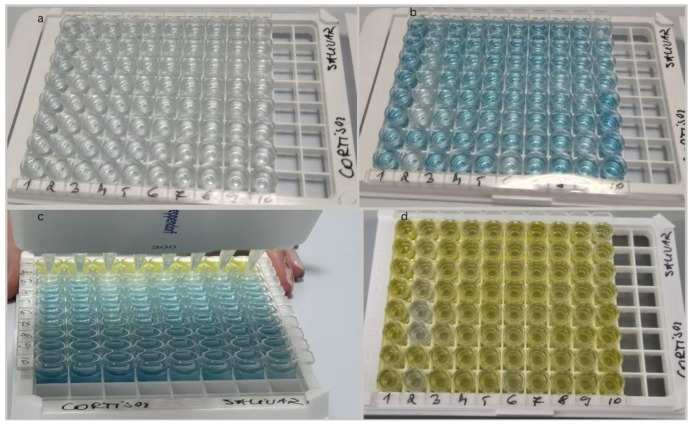
(**a**) The plate before adding the chromogenic substrate; (**b**) the plate after adding the chromogenic substrate; (**c**) the addition of the stopping solution; (**d**) chromogenic reaction turning deep yellow.

**Figure 4:**
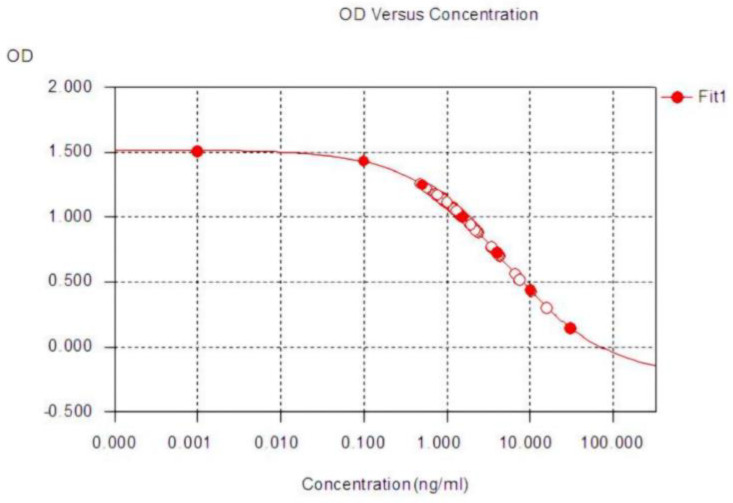
The standard curve.

Sample processing included:

1. Centrifugation—to remove cell debris or precipitates, the unstimulated saliva samples were centrifuged for 5 min at 5400 rpm using the MiniSpin Eppendorf (Eppendorf SE, Eppendorf SE, Germany, 2021) (Figure 1a–c).
2. According to the standardized protocol, controls, samples, and the enzymatic conjugate (cortisol labeled with horseradish peroxidase) were added by pipetting into wells coated with a monoclonal antibody specific to cortisol.
3. Incubation for one hour at room temperature (25 degrees). During incubation, the cortisol present in the standards, controls, or samples competed with the enzymatic conjugate for binding to the monoclonal antibody immobilized at the bottom of the well.
4. After 5 washing cycles, the complete elimination of unbound reagents was ensured (Figure 2).
5. The chromogenic substrate was added, generating a color change proportional to the cortisol concentration in the sample (Figure 3a,b).
6. Final incubation: the plate was incubated again for 30 min at room temperature.
7. Stop reaction: stopping solution was added, which turned the color to a deep yellow (Figure 3c,d).
8. Plate reading: the absorbance of each well was measured using a DYNEX DSX Automated ELISA System spectrophotometer, Dynex Technologies, Chantilly, VA, USA at the specified wavelength (450 nm). The cortisol concentration in the sample was determined by measuring the absorbance of each well and interpolating the values on the calibration curve, with cortisol concentration being inversely proportional to the color intensity in the well.
9. Analysis and interpretation: the standard curve was constructed from the absorbance values of the standards (Figure 4). Cortisol concentration in salivary samples was determined by interpolation using spectrophotometer software, Version 6.25.

All data were entered into a database, and statistical analysis was performed using IBM SPSS software, version 23 (Armonk, NY, USA: IBM Corp), with a confidence interval (CI) set at 95% and a significance level set at 5% (0.05) for the statistical analysis (*p*-value < 0.05). The nonparametric Mann–Whitney test was applied for the comparative analysis of cortisol levels between the two groups, and the nonparametric Kruskal–Wallis test was applied to establish a correlation between the severity of MIH and salivary cortisol levels.

## 3. Results

The study group included 60 patients, comprising 35 girls and 25 boys, aged between 5 and 9 years, with a mean age of 7.2 (standard deviation of 1.09), with no significant differences between the two subgroups. Of the total 31 MIH cases, 14 (45.16%) had a severity grade of 2, 11 (35.48%) had a severity grade of 3, and 7 (22.58%) had a severity grade of 1. In the case group, the mean value of salivary cortisol level was considerably higher compared to the mean value of the control group, the values having no Gaussian distribution, suggesting that the stress level experienced by patients with MIH was higher than that experienced by patients in the control group (2.63306 > 0.95954 ng/mL). Comparative evaluation of salivary cortisol values in the two groups showed statistically significant differences, with a *p*-value of 0.0001 (*p* < 0.005) following the application of the nonparametric Mann–Whitney test.

Mean cortisol values and standard deviation in the two groups are presented in Table 1: (Table 1)

In terms of sex distribution, the mean value was 1.89606 ng/mL in girls and 1.76104 ng/mL in boys, with *p*-value 0.576 (*p* > 0.05), with no statistical significance, although the highest recorded value in the study was among girls (10,129 ng/mL) (Table 2).

To correlate the degree of severity with the salivary cortisol level, the nonparametric Kruskal–Wallis test was applied, which showed statistically significant differences, indicating a progressive increase in cortisol level from grade 0 (MIH absent) to grade 3, with a statistically significant (*p* < 0.05) *p*-value of 0.001 (Table 3). The highest salivary cortisol level (10.129 ng/mL) recorded in this study corresponds to severity grade 3.

The values in the table shows a progressive increase in the mean cortisol level, correlated with the severity of MIH (0–3), according to the Kruskal–Wallis test (*p*-value = 0.001, *p* < 0.05), suggesting that the anxiety experienced by a child patient is proportional to the severity of the enamel defects and objectified by the salivary cortisol level.

## 4. Discussion

MIH is a qualitative enamel developmental defect, of systemic etiology, affecting one or more first permanent molars and sometimes incisors of the same dentition. Clinically, it is manifested by visible opacities, with shades ranging from opaque white to yellow brown, their severity being asymmetrically distributed, the affected teeth being prone to post-eruptive fractures [20,21].

Therapies for this pathology are varied and correlated with the degree of severity, ranging from noninvasive remineralization or sealing therapies, to minimally invasive restorative options with glass ionomer cements or composite resins, to indirect restorations with pedodontic zirconia crowns or even extraction [22,23].

Regardless of the therapeutic option chosen, one of the critical elements of the clinical picture is represented by the specific hypersensitivity of MIH, which may be accompanied by the failure of anesthetic techniques. Thus, by suboptimal pain control during restorative procedures or even during routine check-ups, child patients experience acute episodes of dental fear and anxiety (DFA), requiring holistic therapeutic approaches, including psychological support and personalized interventions, behavioral modulation, optimal pain control, and appropriate restorative therapy [24,25].

In pediatric dentistry, behavior management techniques such as tell–show–do, distraction, and positive reinforcement are often employed to help children feel more comfortable and relaxed during dental procedures [12,13]. These techniques, being indispensable, were rigorously applied in the present study, the patients benefiting from the psycho-emotional comfort necessary for further collaboration [26].

The results of the present study suggest that dentists may incorrectly interpret the noncooperative behavior of pediatric patients with MIH [27] when, in fact, patients who seemingly have difficulty accepting or refuse dental procedures may actually be experiencing psychological stress, objectively reflected in elevated cortisol levels.

In order to develop an appropriate treatment plan that integrates behavioral management, effective pain control, and long-lasting restorative methods, it is essential for practitioners to accurately assess the actual level of dental fear and anxiety as well as the degree of hypersensitivity of children. Currently, the lack of a universal standardized assessment tool is a challenge in the complex management of patients with MIH.

Behavioral analysis tools such as the Wong–Baker Faces Pain Rating Scale and FLACC (face, legs, activity, cry, consolability) index are useful for assessing pain and discomfort, especially in children, but they are not sufficient, nor are they specific or objective; they do not quantify dental hypersensitivity, do not distinguish between dental pain and dental anxiety, and do not provide relevant information for specific treatments for this condition [28,29].

The Wong–Baker Faces Pain Rating Scale is a visual assessment tool commonly used to measure pain intensity in children. It consists of a series of faces displaying different expressions, from a smiling face representing “no pain” to a crying face representing “the most pain”.

This scale is a useful but not sufficient or objective tool for assessing children’s anxiety, being more suitable for illustrating the discomfort perceived by children through the six expressive faces corresponding to different degrees of pain. For a more accurate and objective assessment of stress, it is necessary to complement this with the analysis of a biological parameter such as salivary cortisol levels, which can provide a concrete indicator of the physiological response to stress during office visits [30]. The FLACC index is an essential tool used by dentists and pediatric specialists to objectively assess and communicate the level of hypersensitivity or pain experienced by the patient, especially in children who cannot verbally express discomfort. This score allows a continuous monitoring of the patient’s physiological and behavioral reactions and is crucial for adapting therapeutic strategies during each treatment session [31]. By carefully evaluating each component of the FLACC index, the clinician can adjust the dental approach according to the intensity of pain and discomfort experienced, thus optimizing behavioral management and patient comfort. The use of this tool also helps to reduce the risk of stress and anxiety among patients, ensuring a more efficient and less traumatizing treatment experience.

Stress can be defined as the result of physical and mental reactions generated by our inability to differentiate between real risks and personal expectations. It produces anxiety and can negatively influence the nervous, endocrine, and immune systems. Anxiety is an organic response, manifested both psychologically and physiologically, characterized by somatic, emotional, cognitive, and behavioral conditions [32].

A recent longitudinal study highlights that even a simple professional dental prophylaxis procedure increases salivary cortisol levels in children with behavioral management problems. This finding is supported by our study, which shows that children with MIH, of varying degrees of severity, frequently exhibit behavioral issues, reflected in elevated cortisol levels [33].

Salivary cortisol can also be used as an evaluation tool for other pain-associated conditions in the oro-maxillofacial region, such as temporomandibular disorders (TMD). It has been demonstrated that salivary cortisol levels are reliable for assessing self-reported pain perception in adolescents with and without TMD. The findings showed that adolescents with TMD had higher salivary cortisol levels compared to those without TMD. Additionally, a direct relationship was observed between self-reported pain in the temporomandibular joint area and salivary cortisol levels [34].

Regarding sex distribution, similar results were reported in the study on salivary cortisol levels and early childhood caries, where no significant differences were observed between the examined groups in terms of gender (*p* = 0.943) and age (*p* = 0.060). Similarly, in our study, the mean salivary cortisol level was 1.89606 ng/mL in girls and 1.76104 ng/mL in boys, with a *p*-value of 0.576 (*p* > 0.05), indicating no statistically significant difference. This supports the idea that DFA does not specifically affect a particular age group or sex [35].

Other studies have highlighted the role of salivary cortisol in monitoring stress and anxiety, factors that can influence oral health. For instance, a 2010 study investigated the salivary cortisol response to psychological stress in children with early childhood caries, revealing a significant correlation between these two [36]. More recent research has indicated that salivary cortisol levels are correlated with various oral conditions, such as recurrent aphthous stomatitis, highlighting the importance of monitoring this marker in dental practice [37]. In 2012, the significance of evaluating salivary cortisol in the diagnosis of Cushing’s syndrome was highlighted, particularly in children and individuals for whom stress factors may influence cortisol secretion at the adrenal level [38]. A 2024 study further underscores the utility of salivary cortisol as a noninvasive tool for assessing stress in children during medical procedures, providing valuable insights into how children respond to surgical stress without the need for invasive methods [39]. All these findings are consistent with the results of the present study, which indicate a strong correlation between cortisol levels and the severity of the MIH condition.

Cortisol belongs to the group of glucocorticoid hormones synthesized from cholesterol and secreted by the adrenal cortex, the majority of cortisol in the blood circulating in the form bound by corticosteroid-binding globulin (called transcortin) and albumin, while 5–10% of cortisol remains in free and biologically active form. Although determination of blood cortisol levels is widely used, it can lead to false-positive results due to the additional stress associated with blood collection [40]. Measurement of salivary cortisol is a simpler, safer, and less invasive method of determining active cortisol levels, with a strong correlation with free cortisol in the blood. The salivary cortisol level represents about 70% of the free cortisol in the bloodstream, and saliva is easier to collect than blood, not requiring special equipment or additional patient preparation. One of the main advantages of salivary cortisol is that it can be obtained in the participant’s natural environment, avoiding additional stress. This makes it an innovative approach in acute stress biomarker research, with its simple collection method and wide potential applications [41].

The mean physiologic levels of salivary cortisol in children aged 5–9 years in the morning are in the range 5.7–7.2–32.4–36.9 nmol/L, corresponding to values of 0.2052–0.2592–1.175–1.338 ng/mL. These values are compared to the mean recorded in the control group of the study (0.95954 ng/mL) and to the significantly higher values identified in the cohort of MIH patients (2.63306 ng/mL) [42,43]. These differences underline that dental visits may act as triggers of acute cortisol fluctuations, with a more significant impact in children with MIH compared to healthy children, indicating a deeper level of anxiety among MIH patients and thus imposing the need for a more rigorous behavioral management strategy. This notion is further corroborated by other studies that have demonstrated increased cortisol levels in different pediatric populations undergoing various dental therapeutic interventions [44].

The correlation between severity of MIH and salivary cortisol levels (absent MIH—0.94678 ng/mL, Grade 1—1.43757 ng/mL, Grade 2—1.76693 ng/mL, and Grade 3—4.37536 ng/mL) clearly underlines the link between the severity of the condition and the body’s intensified physiological response, reflected by increased cortisol concentrations. These results suggest that MIH-specific hypersensitivity poses a significant challenge in the therapeutic approach to this abnormality, negatively influencing treatment efficacy and increasing the complexity of behavioral management of patients. Practitioners have reported considerable difficulties in the behavioral management of children with MIH, their anxiety being both justified and objectified by the significant elevation of this biomarker [45,46].

The main causes and mechanisms involved in the development of hypersensitivity, which underlie the behavioral problems among children with MIH, include dentin exposure due to reduced enamel mineralization, increased porosity, and changes in the thermal and mechanical properties of enamel, such as decreased hardness and elastic modulus, increased carbon and carbonate concentrations, and higher protein content [40]. These changes lead to premature breakdown of enamel and exposure of subjacent dentin, favoring increased sensitivity, which contributes to deterioration of oral hygiene and a higher susceptibility to caries.

Detailed and exhaustive knowledge of the local and general clinical picture of patients with MIH is essential for the development of tailored practice guidelines that integrate effective strategies for the management of dentin hypersensitivity and behavioral difficulties associated with DFA specific to this category of patients. In this regard, the use of hydroxyapatite-based desensitizing pastes represents a promising approach to ameliorate dentin sensitivity by rebuilding the enamel microstructure and restoring its structural integrity. In parallel, the promotion of an effective anesthetic protocol, with an emphasis on preemptive administration of analgesics, may contribute to optimal pain control and prevention of acute discomfort associated with dental procedures [47,48].

Furthermore, techniques to reduce anxiety, including the use of effective means of communication and different behavioral therapy techniques adapted to the age and needs of each patient, are crucial to prevent intense emotional reactions and to facilitate cooperation during treatment [49]. These techniques should be correlated with the patient’s psycho-emotional pattern, identifying individual traits, such as fear of dental treatments or heightened emotional sensitivity, in order to implement personalized behavioral management. Thus, therapeutic options can be calibrated according to the particularities of each case, taking into account not only the pathophysiological features of MIH, but also the patient’s behavioral response, to ensure effective treatment and a less traumatic dental experience. This holistic approach will help improve patients’ quality of life and minimize the risks of long-term complications, such as further enamel damage and the development of other associated dental conditions. It facilitates patient-centered care, reducing the need for invasive interventions, such as general anesthesia, thereby helping to optimize costs and improve the accessibility of dental treatments.

The fact that salivary cortisol levels are higher in children with MIH but not in those with other diseases like caries, suggests that the anxiety and stress experienced by MIH patients may not be driven only by pain, as clinically, pain is present in both conditions. Instead, the stress response in MIH children could be linked to a combination of factors, including chronic hypersensitivity, prior negative dental experiences, and even psychological distress associated with esthetic concerns. Moreover, it is worth considering whether cortisol might play a dual role in MIH—both as a contributing factor and as a consequence. Since elevated cortisol levels during early childhood development have been identified in enamel hypomineralization, chronic stress exposure in critical periods could be a potential etiological factor for MIH. This bidirectional relationship may suggests that cortisol is not just a response to MIH-related challenges but may also be an underlying factor in its development, creating a cycle where stress contributes to MIH and MIH exacerbates stress [50,51].

A recent study suggests a potential link between elevated cortisol levels during early childhood and the development of MIH, revealing a significant correlation between stress and MIH, indicating that children with higher stress scores experience a greater occurrence of this enamel defect. The study concluded that stress is potentially one of the main causal factors contributing to MIH development [52].

Moreover, glucocorticoid receptors have been detected on ameloblasts, suggesting that elevated cortisol levels could directly influence the enamel developmental process. This finding implies that stress-induced cortisol secretion during critical periods of tooth development may disrupt normal enamel mineralization, leading to conditions like MIH [53].

The hypothesis that children with MIH have significantly higher levels of salivary cortisol and thus a higher degree of DFA than children without MIH is confirmed by the data provided by the study. These results suggest that MIH is not only pathophysiological but also behaviorally challenging and requires a tailored and integrated therapeutic approach to ensure adequate management of pain, anxiety, and dentin hypersensitivity.

## 5. Conclusions

This study confirms that children with MIH exhibit significantly higher salivary cortisol levels compared to those without MIH, reflecting an increased level of dental fear and anxiety. These findings underscore the critical need for personalized therapeutic strategies aimed at behavioral management and effective pain control to enhance the dental care experience for these patients.

The successful management of hypersensitivity and anxiety in children with MIH requires a multidisciplinary and proactive approach. This includes the implementation of desensitization techniques, preemptive analgesia for optimal pain control, and targeted anxiety reduction methods. By integrating these strategies, clinicians can minimize treatment-related stress, reduce trauma, and create a more comfortable and accessible dental environment for affected children.

## Figures and Tables

**Table 1:** Comparison of cortisol values (ng/mL) between the case and control groups.

MIH	Mean Value	Standard Deviation	Minimum Value	Maximum Value
YES	2.63306	2.065567	1.133	10.129
NO	0.95954	0.330475	0.474	1.922

**Table 2:** Comparison of cortisol levels (ng/mL) between boys and girls.

Sex	Mean Value	Standard Deviation	Minimum Value	Maximum Value
Female	1.89606	1.932252	0.568	10.129
Male	1.83885	1.424165	0.474	7.498

**Table 3:** Salivary cortisol levels (ng/mL) by MIH severity grade.

MIH Severity Grade	Mean Value	Standard Deviation	Minimum Value	Maximum Value
0	0.94678	0.329669	0.474	1.922
1	1.43757	0.418561	1.133	2.095
2	1.76693	0.611382	1.199	3.358
3	4.37536	1.722815	1.809	10.129

## Data Availability

The original contributions presented in the study are included in the article; further inquiries can be directed to the corresponding author.
